# Atypical presentations of mucocutaneous TB in HIV: A case series from South Africa

**DOI:** 10.1016/j.jctube.2025.100524

**Published:** 2025-04-06

**Authors:** Mahlatse Cordelia Kgokolo, Mohlominyane Jeffrey Mokheseng, Jabulile Johanna Makhubele, Shalate Charlotte Siwele, Tinashe Irvin Maphosa, Tsholofelo Kungoane

**Affiliations:** a Department of Dermatology, School of Medicine, Faculty of Health Sciences, University of Pretoria, Private Bag X323, Arcadia 0007, South Africa; b Department of Oral Pathology and Maxillofacial Pathology, School of Dentistry, Faculty of Health Sciences, University of Pretoria, Private Bag X323, Arcadia 0007, South Africa

**Keywords:** HIV, Mycobacteria, Lupus vulgaris, Oral TB, Tuberculosis, Antiretroviral therapy, Mucocutaneous disease

## Abstract

**Introduction:**

Tuberculosis (TB) remains a major burden of disease worldwide, especially in Human immunodeficiency virus (HIV)-infected patients. Cutaneous forms of TB account for approximately 10 % of all extrapulmonary tuberculosis cases, with oral manifestations accounting for less than 1 % of these cases. A high index of suspicion is essential when diagnosing rare clinical presentations. The response to treatment is excellent in most patients, particularly those receiving concomitant, effective antiretroviral (ARV) treatment.

**Patient presentation:**

We report two cases of lupus vulgaris in AIDS patients (CD4 count of 113 cells/mm^3^ and 172 cells/mm^3^, respectively) and one case of mucosal TB in a patient with CD4 count of 365 cells/mm^3^. All the patients were adults referred to the dermatology clinic. The atypical clinical presentations included papules, plaques, and ulcers affecting unusual sites, such as the upper and lower limbs in the case of lupus vulgaris and a tongue ulcer in the patient with oral TB, emphasizing the importance of strong suspicion in HIV-infected patients. All available diagnostic measures were used to confirm the diagnosis.

**Management:**

We demonstrate the importance of multidisciplinary care for patients and an excellent response to anti-TB treatment once diagnosed. We also emphasize the importance of concomitant ARV treatment and patient follow-up.

**Conclusion:**

The outcomes of two of the three patients were good, highlighting the importance of timely clinical diagnosis and treatment, including monitoring and follow-up, while ensuring continued ARV treatment.

## Highlights

• HIV-infected patients present atypical mucocutaneous TB cases.
• Multidisciplinary care enhances treatment outcomes.
• ARV treatment is critical alongside anti-TB therapy.
• A variety of diagnostic tests is key for rare forms of TB.
• Successful management in two out of three cases.

## 1 Introduction

Mucocutaneous tuberculosis (TB) remains an infrequent form of TB presentation worldwide, including in Africa, which reportedly has the highest incidence of HIV/TB coinfection, with South Africa contributing 50 % of cases [1,2]. Cutaneous TB can be classified according to the mode of infection into exogenous and endogenous types. The exogenous types include TB chancre and TB verrucosa cutis. The endogenous types are acquired via contiguity or autoinoculation, which include scrofuloderma, TB orificialis, and lupus vulgaris. Types acquired by hematogenous dissemination include lupus vulgaris, TB gumma, and acute miliary tuberculosis. Tuberculids form an immunological reaction to TB and include papulonecrotic tuberculid, lichen scrofulosorum, and erythema induratum. Bacillus Calmette-Guérin (BCG) vaccination could result in BCG cutaneous abscess [[3], [4], [5]].

Lupus vulgaris generally occurs in individuals with moderate-to-high immunity; however, mucosal TB occurs at low CD4 counts. Lupus vulgaris is the most common morphological variant of cutaneous TB [6] and is typically a chronic and progressive form of cutaneous TB. Clinical presentations include papules or plaques, ulcerative or mutilating, vegetating, and tumor-like or papulonodular lesions, with the most commonly affected sites including the head and neck and, less frequently, the limbs, trunk and buttocks. Variable clinical presentations, however, are expected in immunosuppressed patients [7,8].

Oral TB usually results from secondary bacterial inoculation of oral mucosa breached by any ulceration or minor masticatory trauma, as well as by infected sputum or by hematogenous dissemination from other infected sites [9,10]. Approximately 0.1–0.5 % of patients with pulmonary TB will develop secondary oral TB, most commonly affecting the tongue, followed by the palate, lips, buccal mucosa, and gingiva [9]. It usually presents as nonhealing ulcers but may also occur as nodules, granulomata or fissures or as tuberculous osteomyelitis of the jaw [[9], [10], [11]]. Oral TB ulcers usually present as single rather than multiple lesions and tuberculous ulcers have an indurated, irregular, undermined margin and a necrotic base [12]. Rarely, oral TB may be due to primary infection by direct bacterial inoculation; the most common site is the gingiva, where the primary TB presents as a diffuse erythematous patch or as diffuse gingival enlargement [11,13].

In public health settings in South Africa, we adopt a precautionary and proactive approach to treatment. This approach has resulted in the reduction of TB in HIV patients, nationally. For these patients. We explored all diagnostic measures available at the time in a clinical setting. In contrast to polymerase chain reaction (PCR) and culture, clinical measures, including pathology and imaging, may give quicker results. All our patients were diagnosed based on a combination of important clinical clues.

There is paucity of published data on mucocutaneous TB in HIV. This case series adds important information to the current body of knowledge. We describe three patients with HIV/TB coinfection and atypical clinical presentations. The importance of a high index of suspicion is highlighted, as is the importance of exploring different diagnostic modalities (Table 1). Starting patients on standard anti-TB and antiretroviral (ARV) treatment achieved an excellent response to treatment.

**Table 1:** Clinical presentation and diagnostic results.

	**Case 1**	**Case 2**	**Case 3**
**Sites involved**	Ulcer (Elbow)	Papules and plaques (trunk & limbs)	Ulcer (Tongue)
CD4 count	113 cells/mm^3^	172 cells/mm^3^	365 cells/mm^3^
Viral load	7,940,000 copies/mL	<50 copies/mL	215 copies/mL
Ziehl–Neelsen stain	Positive	Negative	Positive
TB culture	Positive	Not done	Not done
PPD	Not done	Reactive (Fig. 6)	Not done
CXR	Confirmed TB	Fibrotic changes of previously treated TB	Confirmed TB
Skin biopsy for histopathology	Confirmed TB	Confirmed TB	Confirmed TB
TB LAM test in urine	i) Positive	i) Not done	i) Not done
PCR	ii) Positive	ii) Negative	ii) Not done

CD4 cell count- an indicator of immune function in HIV infected patients.

TB- Tuberculosis.

PPD- purified protein derivative – a method used to diagnose TB.

CXR- Chest X-Ray.

TB LAM (lipoarabinomannan) antigen test- a rapid antigen test used to screen for active TB in urine of HIV positive patients, providing results in minutes.

## 2 Case series

### 2.1 Ethical considerations

Ethical approval was obtained from the Faculty of Health Sciences Research Ethics Committee of the University of Pretoria (Ethics reference number: 740/2024). All patients provided informed consent.

### 2.2 Patient 1

The patient was a 32-year-old, HIV-infected woman with a history of abdominal TB, two years previously. She was successfully treated with standard anti-TB treatment for nine months. Simultaneously, the patient had been on ARV treatment but had defaulted soon after initiation.

**Fig. 1a and b:**
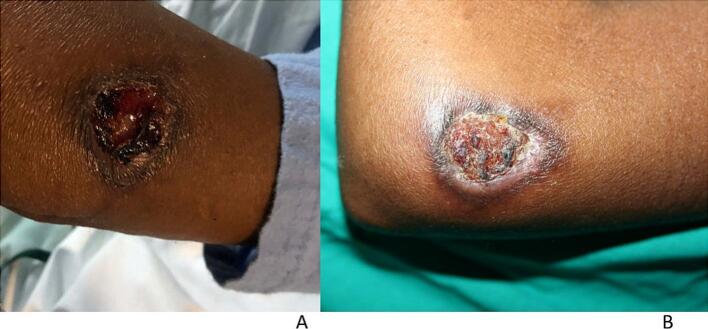
Patient 1: a) The patient presented with a nonhealing ulcer on the left elbow. b) Four weeks after treatment with standard anti-TB treatment and antiretroviral therapy.

She presented to the dermatology clinic with a nine-month history of a nonhealing painful ulcer, which had started as an abscess draining pus, affecting the left elbow (Fig. 1a). The patient was emaciated and ill-looking. The ulcer was 3 × 2 cm, with a raised violaceous border, a hemorrhagic-crusted rim and a granulomatous center. There was no history of trauma or cough. She had a positive TB contact, reporting that she had been living with her uncle, who was newly diagnosed with TB but not yet on treatment. The investigations included a complete blood count, which revealed microcytic, normocytic anemia, a raised C-reactive protein (CRP) of 138 mg/L, negative syphilis serology (TPHA and RPR), negative bacterial and fungal cultures (pus swab), a CD4 count of 113 cells/mm^3^, and a viral load of 7,940,000 copies/mL, positive Gene Xpert MTB/Rif Ultra test with Rifampicin sensitivity (tissue homogenate), and a positive TB lipoarabinomannan (LAM) antigen test (urine).

**Fig. 2a and b:**
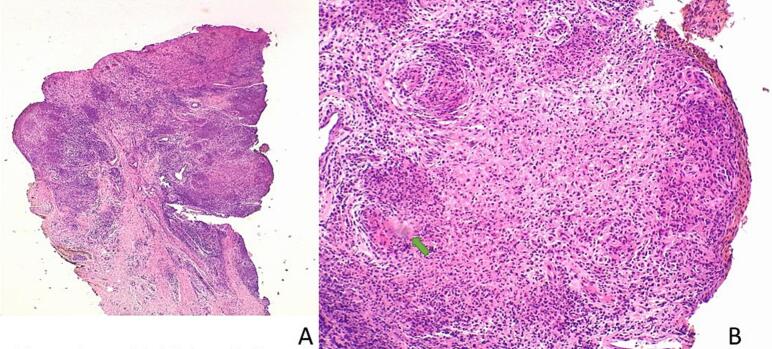
Histopathology of Patient 1 revealing areas of poorly-formed granulomas with caseous necrosis, suggestive of a diagnosis of TB at a) 10x magnification and b) 20x magnification. Hematoxylin and eosin (H&E) staining.

Multiple 4 mm punch biopsies revealed similar changes, with histopathology revealing poorly-formed granulomas with caseous necrosis features suggestive of a diagnosis of TB (Fig. 2a and b). Ziehl–Neelsen staining, TB culture (tissue), and TB PCR (tissue) were positive. Chest X-ray revealed left sided pleural effusion with peri-broncho-vascular thickening, features suggestive of TB.

The final diagnosis was ulcerative Lupus vulgaris with pulmonary TB and HIV coinfection. The patient was started on standard anti-TB and ARV treatment and was referred for wound care. Four weeks of treatment revealed improvement in the ulcer (Fig. 1b), some weight gain, and a clear chest X-ray.

### 2.3 Patient 2

A 48-year-old HIV-infected male patient presented to the dermatology clinic with an eight-month history of asymptomatic papular and plaque lesions increasing in size and number, affecting the arms, trunk and legs. The lesions started six months after the patient had defaulted second-line ARV treatment, which was initiated following the failure of first-line ARV treatment caused by poor compliance at the time. Five years before presenting to the dermatology clinic, the patient had been successfully treated for pulmonary TB with standard anti-TB treatment for six months. The patient was not coughing. Systemic examination revealed nothing of note. Chest X-ray revealed fibrotic changes of previously treated TB.

**Fig. 3:**
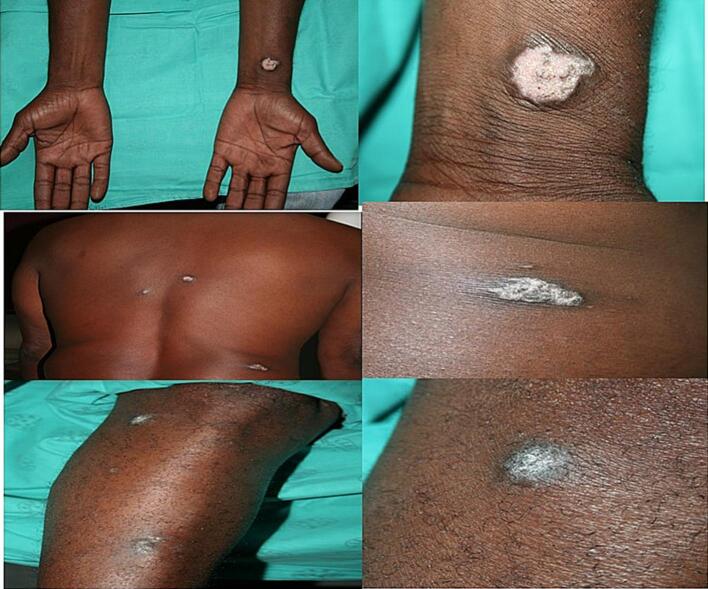
Patient 2 presenting with papules and plaques with a chalky white surface affecting the left wrist area, back, and right lower leg.

**Fig. 4a and b:**
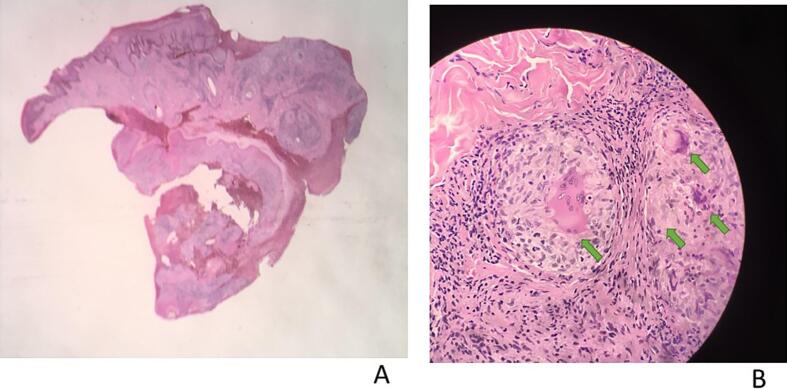
Patient 2. Histopathology showing tuberculoid granulomas with variable caseous necrosis, Langhans and foreign body giant cells, features diagnostic of TB a) 10x magnification and b) 20x magnification. H&E staining.

**Fig. 5a and b:**
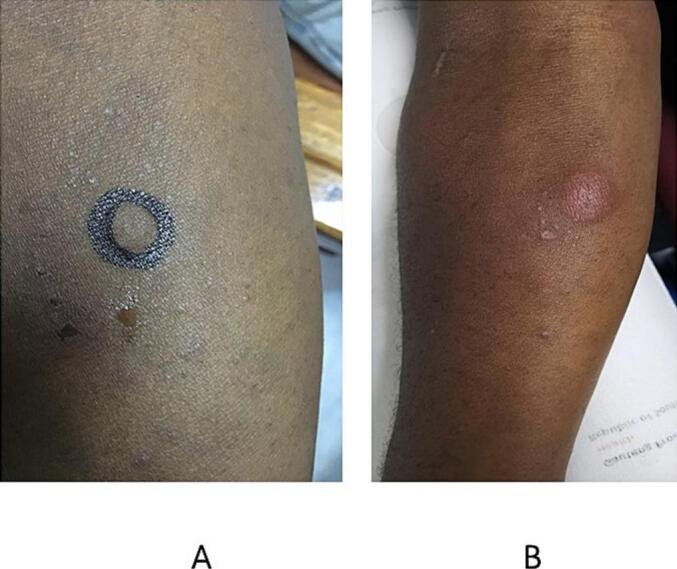
Patient 2 presenting with a highly reactive Mantoux test (20 mm).

The CD4 count was 172 cells/mm^3^, and the viral load was less than 50 copies/mL. Skin examination revealed varying-sized lesions with chalky white overlying surface areas (Fig. 3). Multiple skin biopsies revealed similar changes, with histopathology revealing granulomatous inflammation with variable caseous necrosis, Langhans and foreign body giant cells, cutaneous TB was considered (Fig. 4a and b). Tissue culture was not done. The purified protein derivative (PPD) skin test was highly reactive (20 mm) (Fig. 5 a & b). Ziehl–Neelsen staining and TB PCR were negative. The patient was reinitiated on concomitant standard anti-TB and ARV treatment but was lost to follow-up.

### 2.4 Patient 3

A 56-year-old HIV-infected male patient was referred by oral pathology to the dermatology clinic with a one-year history of a painful ulcer affecting the dorsum of the anterior third of the tongue. He had been on ARV treatment for 21 years. There was no history of TB contact, and the patient had never been treated for TB. There were no constitutional symptoms. Other than generalised lymphadenopathy, the patient looked healthy.

**Fig. 6a and b:**
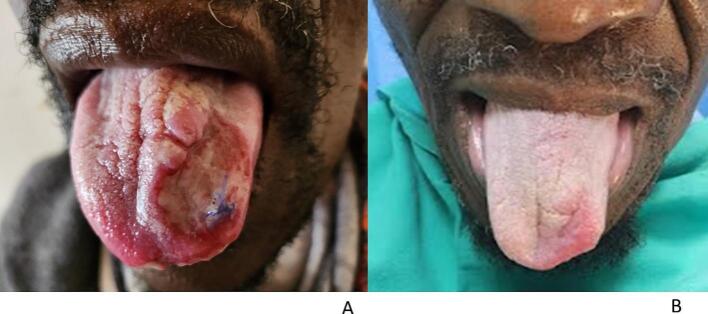
Patient 3, a) presenting with a painful ulcer on the anterior third of the tongue and b) showing almost complete healing six months after treatment.

Examination of the tongue revealed an ulcer affecting the dorsum one-third of the tongue to the left, with rolled erythematous margins and a granulomatous surface with sloughing and creamy whitish areas due to oral thrush (Fig. 6a). The investigations included a full blood count with microcytic normochromic anemia and negative syphilis serology (TPHA), while the CD4 count was 365 cells/mm^3^, and the viral load was 215 copies/mL.

**Fig. 7a and b:**
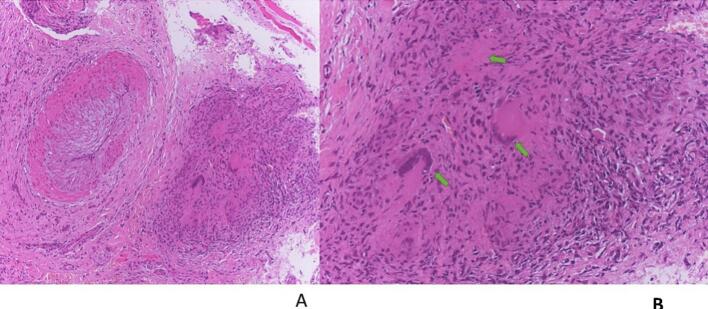
Patient 3. Histopathology showing multiple granulomas with caseous necrosis and Langhans giant cells suggestive of TB at a) 10x and b) 20x magnification. H&E staining.

An incisional ulcer biopsy was performed, with multiple well-formed granulomas composed of epithelioid histiocytes and Langhans giant cells with caseous necrosis revealed on histopathological examination; these granulomas are diagnostic features of TB (Fig. 7a and b). Ziehl–Neelson stain was positive. Tissue culture was not done. Chest X-ray revealed hilar lymphadenopathy and miliary opacities. Our final diagnosis was orificial TB with pulmonary TB and HIV coinfection. The patient was started on standard anti-TB and concomitant ARV treatment, fluconazole, and miconazole oral gel. Six months after initiating treatment, the ulcer showed remarkable improvement, with almost complete healing (Fig. 6b). Oral candidiasis had also completely healed. The chest X-ray was clear.

## 3 Discussion

Compared with HIV-negative patients, HIV-infected patients are at greater risk of reactivation of latent TB, the development of new TB infections, or the rapid progression of active TB [[14], [15], [16]]. There is a synergistic relationship between TB and HIV infection, with each accelerating the progression of the other, with HIV-infected patients with TB having shorter survival than TB-free HIV-infected patients with comparable CD4 counts [15].

Our first patient, in addition to being at risk of direct contact with active TB and not being on treatment, had been previously successfully treated for abdominal TB, confirming the possibility of reinfection or reactivation via hematogenous spread. She was found to have Lupus vulgaris in the clinical setting of pulmonary TB. Her CD4 count was very low, and her viral load was very high.

Similarly, our second patient had previously been treated for pulmonary TB but developed Lupus vulgaris in the absence of active pulmonary TB. Notably, he had a low CD4 count and low viral load, but the Mantoux test showed high reactivity. The commonality between the two patients with Lupus vulgaris was that both had defaulted ARV treatment and both had AIDS, as defined by low CD4 counts. HIV infection renders patients more susceptible to developing TB and lowers treatment success rates in these patients. The treatment success rate among coinfected patients is 77 %, whereas it is 86 % (for first-line TB regimens) for those with TB only [2]. Moreover, the rapid development of drug-resistant TB strains is contributing to poor treatment outcomes, further complicating treatment efficacy [14].

Our last patient had no TB contact and had not been previously treated for TB but had developed oral TB in the presence of pulmonary TB. His CD4 count was 365 cells/mm^3^, and his viral load was low but slightly more than 200 copies/mL, indicating incomplete viral suppression. The patient had been on first-line ARV treatment for 21 years. The burden of HIV/TB coinfection in South Africa necessitates the exploration of differential diagnoses.

This case series presents strong histopathological evidence confirming granulomatous inflammation with caseous necrosis in all three cases and typical Langerhans giant cells in two of the three cases. Various investigations were conducted to confirm the diagnoses. Concerning Lupus vulgaris, the patient with low CD4 count and high viral load had a positive TB culture and Ziehl–Neelsen stain. In contrast, the patient with both low CD4 count and viral load was confirmed via a highly reactive PPD; however, the Ziehl–Neelsen stain was negative, and TB culture was not performed (Table 1).

The diagnosis of Lupus vulgaris depends on the patient’s history, clinical examination and suggestive histopathological findings with or without the demonstration of acid-fast bacilli via Ziehl–Neelsen staining. A highly positive PPD (Mantoux test) may provide additional diagnostic support [6]. In Lupus vulgaris, acid-fast bacilli are not always identified, including in patients with normal functioning immunity. In a series of 10 cases by Marcoval et al., no acid-fast bacilli were detected [7]. Visser and Heyl [17] demonstrated acid-fast bacilli in three out of 16 biopsies. PCR for M. tuberculosis DNA yielded positive and negative results in Lupus vulgaris lesions [18].

Little is known about the occurrence of mucocutaneous TB in HIV-infected patients despite the high burden of HIV/TB coinfection in South Africa [19,20]. In cases where clinical and histopathological findings suggest Lupus vulgaris but the Ziehl–Neelsen stain, culture and PCR results are negative, a trial of anti-TB treatment may be worthwhile [21,22]. Two of our three patients responded well to treatment.

In the public health setting in South Africa, patients are often diagnosed based on available clinical clues, which include the history of TB treatment or exposure, clinical presentations, and available tests in the background of HIV coinfection. These patients are initiated on standard anti-TB treatment, continued on ARV treatment and monitored for response at follow-up.

## 4 Conclusion

Mucocutaneous TB in the setting of high HIV prevalence requires a high index of suspicion to be accurately diagnosed. Multiple diagnostic tools should be used to make a diagnosis. Once a diagnosis has been confirmed, concomitant anti-TB treatment, ARV treatment, and monitoring are essential.

## CRediT authorship contribution statement

**Mahlatse Cordelia Kgokolo:** Writing – review & editing, Writing – original draft, Supervision, Project administration, Conceptualization. **Mohlominyane Jeffrey Mokheseng:** Writing – review & editing. **Jabulile Johanna Makhubele:** Writing – review & editing. **Shalate Charlotte Siwele:** Writing – review & editing. **Tinashe Irvin Maphosa:** Writing – review & editing. **Tsholofelo Kungoane:** Investigation.

## Declaration of competing interest

The authors declare that they have no known competing financial interests or personal relationships that could have appeared to influence the work reported in this paper.
